# Targeted next-generation sequencing for respiratory infections in patients with haematological malignancies

**DOI:** 10.3389/fcimb.2025.1678858

**Published:** 2025-11-19

**Authors:** Jiayu Huang, Su Li, Chuanhe Jiang, Luxiang Wang, Zengkai Pan, Zilu Zhang, Jun Zhu, Wei Chen, Xiaoxia Hu

**Affiliations:** 1 State Key Laboratory of Medical Genomics, Shanghai Institute of Hematology, National Research Center for Translational Medicine, Shanghai Rui Jin Hospital, Shanghai Jiao Tong University School of Medicine, Shanghai, China; 2 Collaborative Innovation Center of Hematology, Shanghai Jiao Tong University School of Medicine, Shanghai, China; 3 GoBroad Medical Institute of Hematology (Shanghai Center), Liquan Hospital, Shanghai, China; 4 Department of Pulmonary and Critical Care Medicine, Shanghai Rui Jin Hospital, Shanghai Jiao Tong University School of Medicine, Shanghai, China; 5 Institute of Respiratory Diseases, Shanghai Jiao Tong University School of Medicine, Shanghai, China

**Keywords:** targeted next-generation sequencing, metagenomic next-generation sequencing, diagnostic efficiency, conventional microbiological tests, treatment efficacy

## Abstract

**Background:**

Patients with haematological malignancies are immunocompromised and prone to respiratory infections, but identification of causative pathogens is challenging. The aim of this study was to analyse the ability of targeted next-generation sequencing (tNGS) to detect pathogens in immunocompromised patients.

**Methods:**

tNGS and conventional microbiological tests (CMT) were performed on samples from the respiratory tract of 99 patients with suspected respiratory infections. Metagenomic next-generation sequencing (mNGS) was conducted in parallel in 43 patients. Comparative analysis was conducted using the Pearson χ2 test and Fisher’s exact test, as appropriate.

**Results:**

The overall microbial detection rates for tNGS were 100% (23/23) in the upper respiratory tract and 96.1% (99/103) in the lower respiratory tract. Microorganism colonization was detected by tNGS in 80.8% (97/120) of cases. The sensitivity of tNGS was approximately 30% higher than that of CMT (87.7% vs. 52.5%; *P* < 0.001), but tNGS had a lower specificity (33.3% vs. 83.3%; *P* = 0.242). tNGS improved the overall treatment success rate by 69.7% (69/99 cases) in CMT true-negative or CMT-partially matched cases. In the paired respiratory tNGS and mNGS cases, tNGS verified 73.3% (11/15) cases of infection, while mNGS only verified 40% (*P* = 0.139).

**Conclusions:**

Most immunosuppressed patients are colonized by microorganisms, and require prompt identification of the cause of any infections. tNGS has promising diagnostic potential and offers valuable information for optimizing antibiotic therapy, especially when compared to CMT.

## Introduction

Patients with haematologic malignancies undergoing chemotherapy or haematopoietic stem cell transplantation (HSCT), whether autologous or allogeneic, are immunocompromised and therefore more susceptible to infections (Neofytos, 2019; Shah et al., 2023). The respiratory tract is the most common site of infection, followed by the mucosa, gastrointestinal tract, and peripheral blood (Xu et al., 2023). Complications from respiratory infections are a major cause of mortality, particularly if inappropriate antibiotic treatment is administered (Periselneris and Brown, 2019). Prompt and accurate pathogen identification is, therefore, critical for optimizing antibiotic therapy.

Conventional microbiological tests (CMT), including (1, 3)-β-D-glucan (G)-, galactomannan (GM) tests, and cultures of blood, sputum, and bronchoalveolar lavage fluid (BALF), are commonly used for infection identification (Periselneris and Brown, 2019). In neutropenic patients, however, >50% of pathogens remain unidentified through CMT (Huang et al., 2023). Additionally, typical imaging findings, though highly suggestive of specific pathogens, rely heavily on the expertise of radiologists. Moreover, procalcitonin (PCT), C-reactive protein (CRP), G, and GM tests can all be influenced by noninfectious factors, making them nonspecific for pathogen identification (Huang et al., 2023). While *in vitro* culture methods can identify bacteria or fungi, they are time-consuming and suffer from low sensitivity (Flournoy, 1994).

The application of metagenomic next-generation sequencing (mNGS) technologies in managing suspected infections is increasing. mNGS can identify novel pathogens, improving diagnostic efficiency and accuracy (Diao et al., 2022); however, its ability to detect RNA viruses is limited, requiring multiple extractions, and its clinical use is often constrained by high costs. Despite its capability to detect a broad spectrum of microorganisms, mNGS readouts necessitate rigorous clinical interpretation to distinguish causative pathogens. Targeted NGS (tNGS), based on a predefined panel of 198 respiratory pathogens, has shown promise in overcoming the aforementioned issues and detecting respiratory infections in immunocompetent patients (Chao et al., 2020; Sibandze et al., 2022), but its feasibility and accuracy in immunocompromised patients, especially with haematologic malignancies, is unclear. In this study, we analysed the ability of tNGS to detect pathogens in this patient population.

## Materials and methods

### Study design and data collection

The records of patients who underwent respiratory tract tNGS for suspected infections at the Shanghai Ruijin Hospital, Shanghai Zhaxin Hospital and Shanghai Liquan Hospital between November 2022 and May 2023 were retrospectively reviewed. The inclusion criteria were as follows: 1) patients aged ≥12 years, and 2) patients with haematologic disorders who received chemotherapy or HSCT, and 3) patients who underwent tNGS testing as a result of the manifestation of symptoms including fever and cough. The exclusion criteria were as follows: 1) asymptomatic patients who underwent tNGS screening for pathogens following prior pulmonary infections; 2) patients who underwent repeated tests with the same type of sample within 7 days during the same febrile episode; and 3) patients who underwent follow-up tNGS tests after anti-infection therapy. Collected data included patient demographics, disease diagnosis, therapies, and clinical diagnosis (infection or non-infection). All procedures complied with the tenets of the *Declaration of Helsinki*. The requirement for written informed consent was waived, and was approved by the Institutional Board of Ruijin Hospital, Shanghai Jiaotong University School of Medicine, and registered at ClinicalTrials.gov (NCT 06708130).

Patients received empirical treatment concurrently when having tNGS test. Owing to respective design, we did not have a uniform criterion for the refinement techniques. Whether a patient receives mNGS is at the discretion of treating physicians. In this study, a total of 26 patients underwent blood mNGS testing, with fever also identified as clinical manifestation. Additionally, 18 patients received respiratory tract mNGS testing, where cough and expectoration were noted as the predominant clinical symptoms. Both methodologies were aligned within 7-day of the same clinical illness episode.

### NGS and CMT

The target species covered by the tNGS panel are listed in **Supplementary Table S1**. Respiratory tract samples with high viscosity were diluted 1:1 with 0.1 M dithiothreitol prior to nucleic acid extraction. Automated nucleotide extraction was performed using a MagPure Pathogen DNA/RNA Kit B (Magen Biotechnology, Guangzhou, Guangdong, China) on a KingFisher™ Flex Purification System (Thermo Fisher Scientific, Waltham, MA, USA). Nuclease-free water (Invitrogen, Waltham, MA, USA) was used as an NTC (non-template control) to detect contamination. The RNA samples were transcribed to cDNA and mixed with genomic DNA as input. cDNA synthesis, multiplex polymerase chain reaction (PCR) preamplification of target loci, and library preparation were performed using the RP100TM Respiratory Pathogen Microorganisms Multiplex Testing Kit (KingCreate Biotechnology Co.,Ltd., Guangzhou, China). The generated libraries were quantified using an Equalbit DNA HS Assay Kit (Vazyme Biotech, Nanjing, Jiangsu, China) with an Invitrogen™ Qubit™ 3.0/4.0 (Thermo Fisher Scientific, Waltham, MA, USA) fluorometer to ensure that all the samples had a library density ≥ 0.5 ng/μL or were subjected to library reconstruction. DNA fragment analysis was performed using the Qsep100 Capillary Electrophoresis System™ (BiOptic. Inc., Jiangsu, China) and its compatible Standard Cartridge Kit. After library qualification, sequencing was performed using the KM MiniSeqDx-CN Sequencing Kit on KM MiniSeq Dx-CN Platform (KingCreate, Guangzhou, Guangdong, China). The sequencing data generated were analysed using a customized bioinformatic workflow. The raw sequencing data were subjected to a quality control procedure. Fastp v0.23.2 was employed for adaptor trimming and low-quality filtering using default parameters, ensuring a minimum of 1 million clean reads with over 85% of them having a Q30 quality score. For a specific pathogen or group, a normalized matched read count that reached RPhK ≥ 10 (matched reads per 100,000 reads, the cut-off determination based on experience) was considered positive.

For the mNGS test, nucleic acid was extracted from peripheral blood, BALF, or sputum samples. Before delivery to the laboratory, the samples were stored at -4 °C. After adding the adapters, PCR amplification DNA libraries were constructed. The quality of the library was evaluated by quantitative PCR. After removing low-quality and short reads and human host sequences, the remaining data were aligned to those of the microbial genome database obtained from the NCBI RefSeq and GenBank databases.

CMT was performed for all events, including smear microscopy, culture, G test, GM test and quantitative real-time PCR. Viruses including cytomegalovirus, Epstein-Barr virus (EBV) and herpes simplex virus-1 (HSV1), were detected via the quantitative real-time PCR.

### Reporting of NGS results

We used institutional electronic medical records and clinical databases to obtain the required information. As a crucial component of this study, an independent committee, comprising two haematologists, one respiratory doctor, and one radiologist conducted an evaluation of the composite clinical diagnosis through partial blinding. Specifically, the committee classified cases as clinically significant infections, infections without clinical significance, or immune events after comprehensive consideration of clinical symptoms, laboratory and radiologic readouts, as well as treatment responses independently without the information of NGS results. And then, the committee members performed a separate evaluation of the tNGS readouts. However, in their review of the final causative pathogen, the committee employed the reference of NGS results, particularly in cases of negative CMT results. Treatment success was defined as antibiotics within 7 days following different detection approaches issued that led to resolution of clinical symptoms.

Due to a lack of standard criteria for interpreting tNGS data, microorganisms (by specimens) were classified into three types based on previous reports leveraging healthy cohorts (Caroll et al., 2019): (A) microorganisms detected with a prominent ability to cause infection or are common causes of pneumonia; (B) microorganisms detected with the potential to cause infection but not commonly associated with infection, as evaluated by clinical experts based on medical records; or (C) microorganisms considered colonizing microbes or microecological flora of the lower respiratory tract, not likely causes of infection. The criteria for mNGS tests were consistent with those of a previous report (Huang et al., 2023).

The NGS results were ultimately classified according to the following criteria: Definite, possible, unlikely, false negative, and true negative (1). Definite: microbes detected as A type by tNGS or detected as positive by mNGS that were consistent with those identified by CMT or specialized causative pathogens. (2) Possible: microorganisms detected as A or B types by tNGS or detected as positive by mNGS that showed potential to cause infection. (3) Unlikely: microbes detected as A or B types by tNGS or positive by mNGS that were non-causative pathogens. (4) False negative: In cases diagnosed with infection, the mNGS or tNGS result was negative, or tNGS detected microbes as C type. (5) True negative: if the NGS result was negative or microbes were detected as C type by tNGS, and the case was diagnosed with non-infection. In definite and possible situations, NGS results were considered true positive, while in unlikely situations, they tended to be false positive. The PPV and NPV were calculated as the ratio of true positive or negative detections to all accrual tNGS readouts. A “complete match” was defined as all microbes identified through different detection methods matching the pathogens of the final clinical diagnosis. A “partial match” was defined as at least one but not all microbes identified matching the pathogens. “Not matched” was defined as all microbes identified differing from the pathogens identified in the final clinical diagnosis.

### Statistical analysis

IBM SPSS Statistics version 25.0 (IBM Corp., Armonk, N.Y., USA) and STATA 17 software were used for the statistical analysis. Continuous and categorical variables are presented as medians (ranges) and counts (percentages), respectively. The sensitivity, specificity, PPV, and NPV were calculated according to previous definitions (Blauwkamp et al., 2019). Comparative analysis was conducted with the Pearson χ2 test and Fisher’s exact test for discrete variables, where appropriate. Test concordance was analysed using the kappa statistic. A *P* value <0.05 was considered to indicate statistical significance.

## Results

### Patient characteristics and sample collection

We performed 142 tNGS tests on 100 patients between November 2022 and May 2023. Of these, three tests were conducted for pathogen screening prior to therapy, five tests were repeated from the same samples within 7 days, and eight tests were follow-up tests after anti-infection therapy. Consequently, 126 tests from 99 patients, with 120 clinical events, were included in the data analysis (**Supplementary Figure S1**). Sixty-four patients (65.7%) underwent allo-HSCT (**Table 1**). The tNGS test samples were divided into two groups: Upper respiratory tract (oropharyngeal swab, group I, 23 cases) and lower respiratory tract[group II, 103 cases, sputum (n=91) and bronchoalveolar lavage fluid(BALF, n=12)]. The most common source of mNGS specimens was peripheral blood (26 cases), followed by BALF (16 cases), pleural effusion (1 case) and sputum (1 case).

**Table 1:** Baseline characteristics of 99 patients.

Characteristics		Total
		n (%)
Age in years *[Median(range)]*	53(13-75)	n/a
Sex	Male	52(52.5)
	Female	47(47.5)
Underlying diseases	AML	40(40.4)
	MDS	14(14.1)
	ALL/LBL	13(13.1)
	CML/CMML	3(3.0)
	MM	13(13.1)
	NHL	13(13.1)
	AA	2(2.0)
	Other diseases	1(1.0)
Therapeutic approach for underlying diseases	Allo-HSCT	65(65.7)
	Auto-HSCT	14(14.1)
	Chemotherapy	20(20.2)

allo-HSCT, allogeneic haematopoietic stem cell transplantation; auto-HSCT, autologous haematopoietic stem cell transplantation; AML, acute myeloid leukaemia; MDS, myelodysplastic syndrome; ALL, acute lymphoblastic leukaemia; LBL, acute lymphoblastic lymphoma; CML, chronic myelogenous leukaemia; CMML, Chronic myelomonocytic leukaemia; NHL, non-Hodgkin’s lymphoma; AA, aplastic anaemia; MM, multiple myeloma; and n/a, not applicable.

### Microbial landscape detected with tNGS

**Figure 1:**
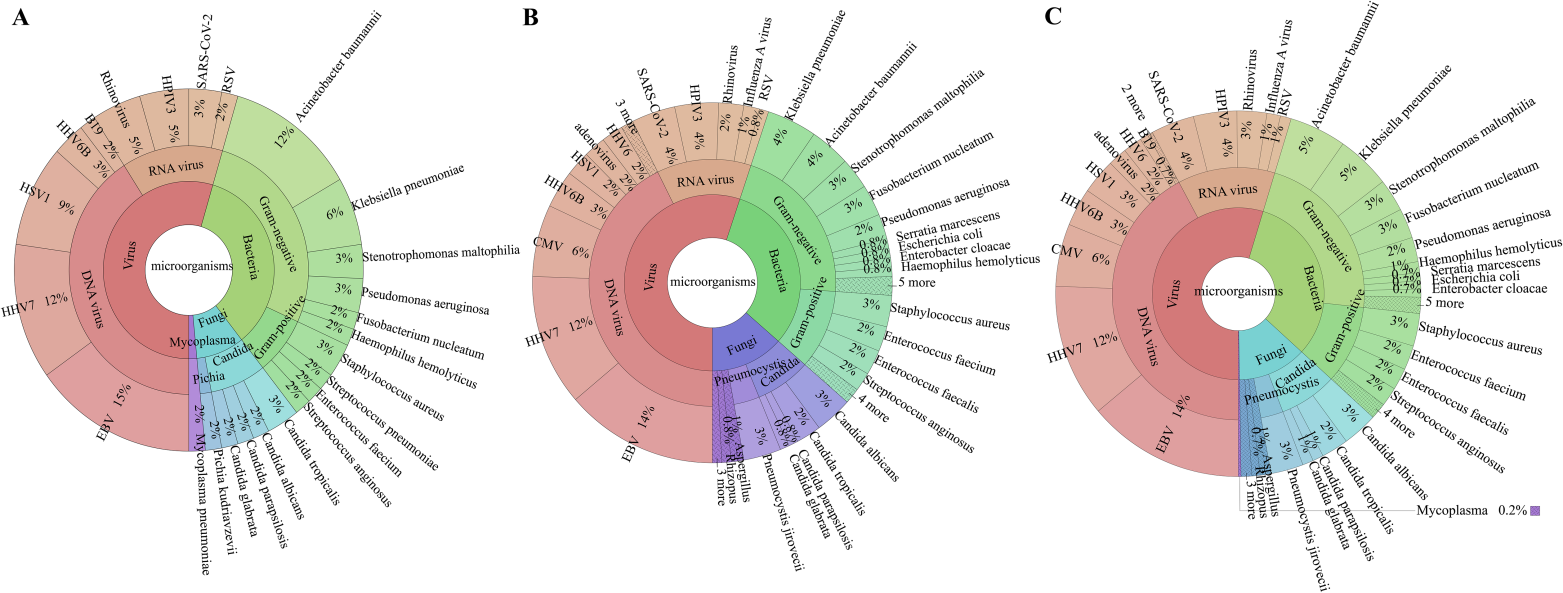
Specific pathogen map. In **(A)** (Group I: upper respiratory tract), the colour green indicates the detection of bacteria, red signifies the identification of viruses, cyan denotes fungi detection, and purple represents the identification of mycoplasma pneumoniae. In **(B)** (Group II: lower respiratory tract), the colour green indicates the detection of bacteria, red signifies the identification of viruses, and purple represents the identification of fungi. “3 more, 4 more, 5 more” in B represents more than three types microorganisms with low detection rate. In **(C)** (combined respiratory sample), the colour green indicates the detection of bacteria, red signifies the identification of viruses, and purple represents the identification of fungi. “3 more, 4 more, 5 more” in B represents more than three types microorganisms with low detection rate. HSV1, herpes simplex virus-1; HHV6B, human herpesvirus 6B; B19, human parvovirus B19; HPIV3, human parainfluenza virus 3; RSV, respiratory syncytial virus; EBV, Epstein–Barr virus; HHV7, human herpesvirus 7; CMV, cytomegalovirus; HHV6, human herpesvirus 6.

The overall microbial detection rate for the tNGS test was 100% (23/23) in group I and 96.1% (99/103) in group II, with 97.8% (89/91) in sputum specimens and 83.3% (10/12) in BALF specimens. The constituent ratios of detected pathogens were similar between group I and group II (**Supplementary Figure S2**). The most common micro-organisms detected by tNGS in both groups were EBV, human herpesvirus (HHV)-7, and *Acinetobacter baumannii* (**Figure 1**).

We conducted paired tNGS tests, defined as tNGS performed on different specimens for the same clinical event, on six pairs. We found that the tNGS results from different specimens, based on the final classification criteria, were mostly consistent, with a concordance rate of 83.3% (**Table 2**).

**Table 2:** The detailed information of paired tNGS tests.

Case	Paired samples (S1-S2)	Pathogens of S1	Pathogens of S2	Clinical events
Case 1	Oropharynx-BALF	Acinetobacter baumannii [C]	Negative	No infection
Case 2	Sputum-BALF	Stenotrophomonas maltophilia [B]	HPIV3 [A]	Pulmonary polymicrobial infection (Fungi and HPIV3)
		Pneumocystis jirovecii [B]		
		HSV1[A]		
		HPIV3[A]		
		EBV[C]		
Case 3	Sputum-BALF	Pseudomonas aeruginosa [A]	Pseudomonas aeruginosa [A]	Pulmonary polymicrobial infection (Bacteria and SARS-CoV-2)
			Candida [B]	
		Candida [B]	CMV [B]	
			SARS-CoV-2 [A]	
Case 4	Sputum-BALF	Klebsiella pneumoniae [A]	HBoV1 [A]	Respiratory tract polymicrobial infection (Bacteria and HBoV1)
		HHV7 [C]		
		HHV6B [C]		
		EBV [C]		
		CMV [B]		
		HBoV1 [A]		
Case 5	Oropharynx-sputum	Pseudomonas aeruginosa [B]	Pseudomonas aeruginosa [A]	Pulmonary and oral mucosa polymicrobial infection (Bacteri, HSV1 and HPIV3)
		EBV [C]	EBV [C]	
		HSV1 [A]	HSV1 [A]	
		HPIV3 [A]	HPIV3 [A]	
		HHV7 [C]	HHV7 [C]	
Case 6	Oropharynx-sputum	Pichia kudriavzevii [B]	Pneumocystis jirovecii [B]	Pulmonary microbial infection (Bacteria and fungi)
			CMV [B]	

BALF, bronchoalveolar lavage fluid; tNGS, targeted next-generation sequencing; HHV7, human herpesvirus 7; HHV6B, human herpesvirus 6B; CMV, cytomegalovirus; EBV, Epstein Barr virus; HSV1, herpes simplex virus1; HRIV3, Human parainfluenza virus type 3; HPIV3, Human Parainfluenza Virus Type 3; HBoV1, Human Bocavirus 1; and SARS-CoV-2, Severe Acute Respiratory Syndrome Coronavirus 2.

A, B, C, three types microorganisms (by specimens) classified based on previous reports leveraging healthy cohorts in Methods (Caroll et al., 2019).

**Figure 2:**
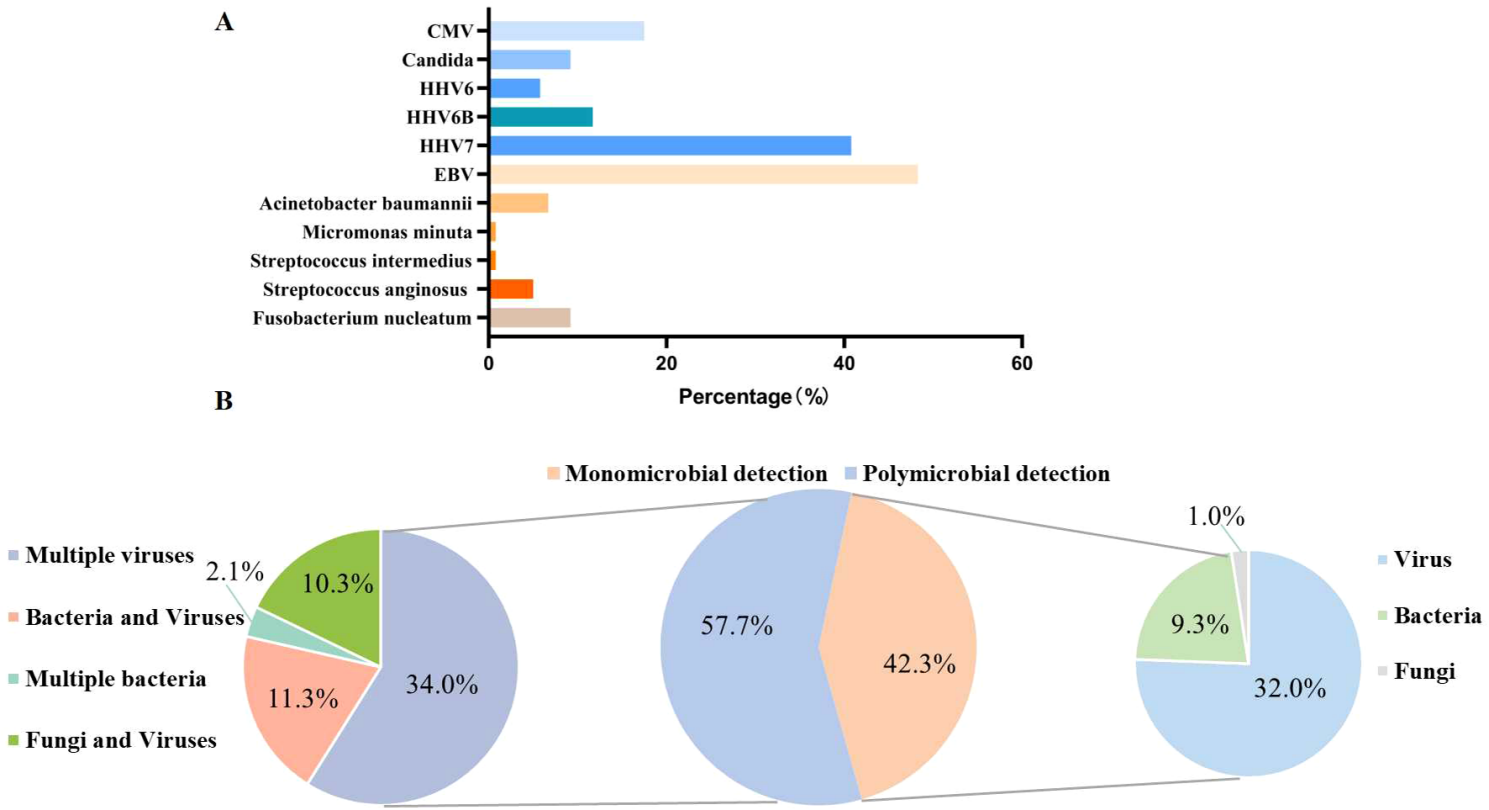
Colonization of microorganisms detected via tNGS across all samples. **(A)** The specific colonizing microorganisms detected. **(B)** The distribution of the identified colonizing pathogens. CMV, Cytomegalovirus; HHV6, Human herpesvirus 6; HHV6B, Human herpesvirus 6B; HHV7, Human herpesvirus 7; EBV, Epstein–Barr virus.

After excluding six paired tNGS cases, we analysed a further 120 cases. Here, tNGS detected microbial organisms in 75% (90/120) of cases (**Figure 2a**). Polymicrobial colonization was detected in 57.8% of patients (n = 52), and monomicrobial colonization was detected in 42.2% of patients (n = 38). Among the polymicrobial organisms colonization, multiple viruses were detected in 34.4% (n = 31), bacteria and viruses in 12.2% (n = 11), fungi and viruses in 8.9% (n = 8), and multiple bacteria in 2.2% (n = 2) (**Figure 2b**).

### False-positive and false-negative tNGS results

Among the patients diagnosed with a clinically significant infection, 14 (12.2%) were missed by tNGS. Of these, 11 cases were not consistent with the pathogen identified in the clinical diagnosis, including colonization (7/11) and latent infection (4/11), and the remaining three cases were negative by tNGS. Among six patients diagnosed as non-infected by the treating physician, four cases tested positive with the tNGS test (**Supplementary Table S2**).

### Comparison of tNGS and CMT

We found that tNGS demonstrated a superior detection rate compared to CMT for viruses, bacteria, *Pneumocystis jirovecii*, and *Candida* (*P* < 0.005, **Supplementary Figure S3a**). The detection rates for *Acinetobacter baumannii*, *Klebsiella pneumoniae*, and *Staphylococcus aureus* differed significantly between the two approaches. In addition, *Klebsiella oxytoca*, *Enterobacter aerogenes*, *Staphylococcus hemolyticus*, *Staphylococcus epidermidis*, and *Aspergillus niger* were only detected by CMT (**Supplementary Figure S3b**).

**Figure 3:**
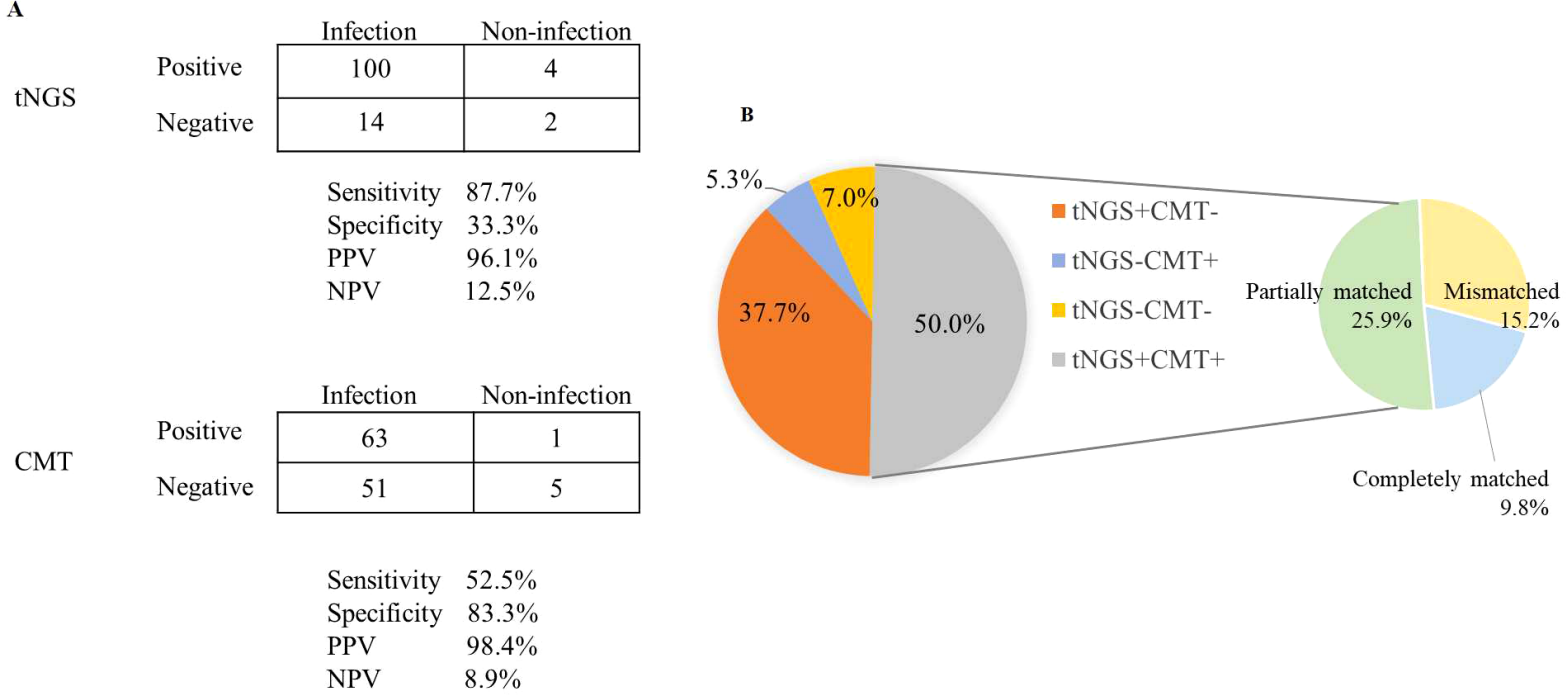
Diagnostic comparison of microorganisms detected via tNGS and CMT. **(A)** A contingency table presented in a 2 × 2 format illustrating the respective diagnostic performance of tNGS and CMT in differentiating infections. **(B)** The consistency of positive results for tNGS and CMT across all samples.

The sensitivity of tNGS was approximately 30% higher than that of CMT (87.7% vs. 52.5%; *P* < 0.001), but tNGS had a lower specificity (33.3% vs. 83.3%; *P* = 0.242; **Figure 3a**). The negative predictive value (NPV) and positive predictive value (PPV) of tNGS were 12.5% and 96.1%, respectively, with a negative likelihood ratio of 0.37 and a positive likelihood ratio of 1.32.

Among the 114 cases diagnosed with clinical infections, 43 (37.7%) had true-positive tNGS results but no corresponding true-positive CMT results, and six (5.3%) had true-positive CMT results without true-positive tNGS results. A total of 57 (50%) patients were detected as true-positive by both tNGS and CMT, and eight (7.0%) were true-negative by both methods. For double-positive samples, the results were completely matched in 11/57 cases and mismatched in 17/57 cases. Most of the mismatched cases (25.9%) were considered “partially matched,” meaning at least one type of pathogen was detected by both tNGS and CMT (**Figure 3b**).

**Figure 4:**
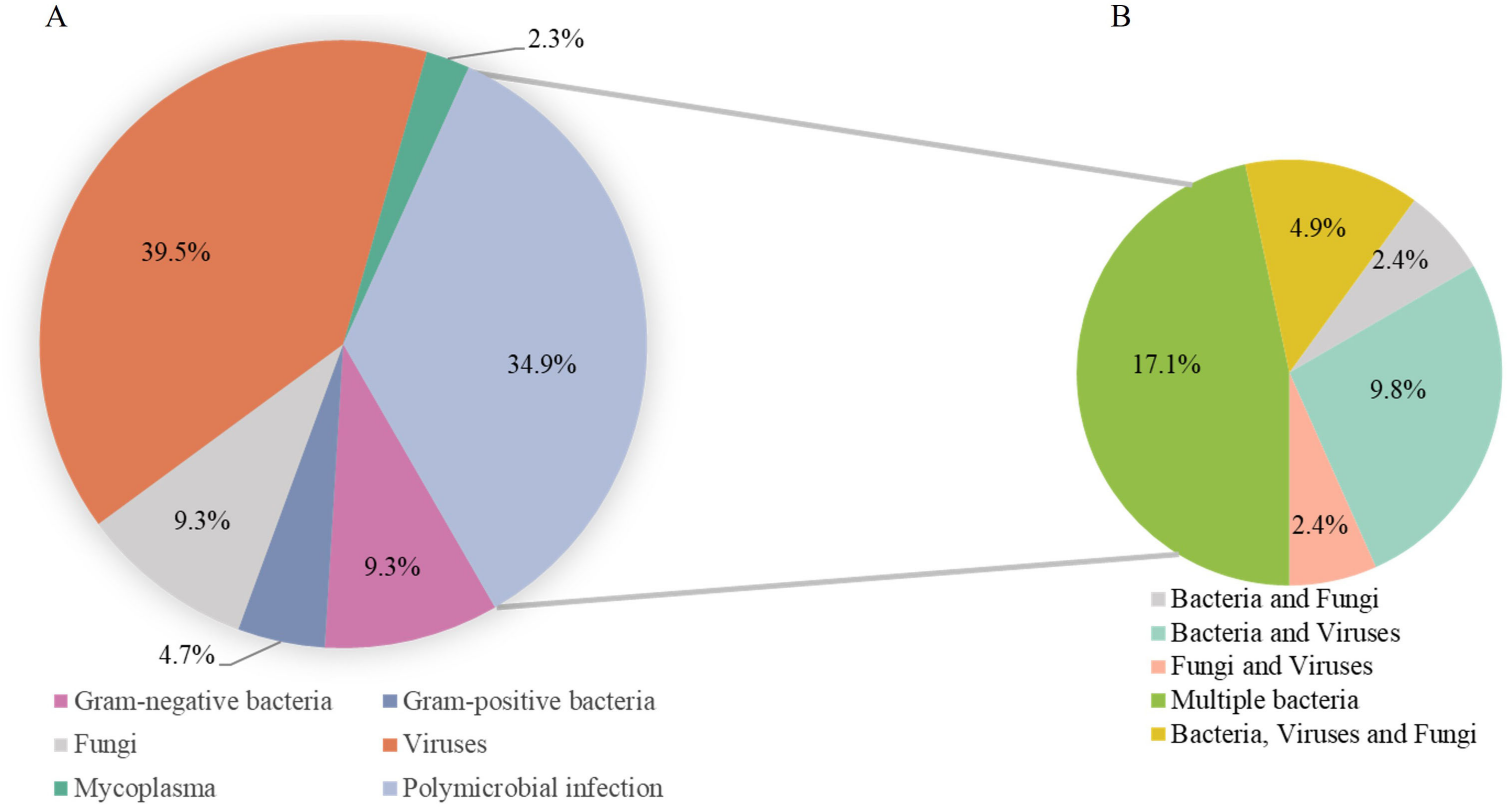
Infection verification by tNGS in CMT-negative patients. **(A)** Pathogens identified through tNGS in cases that tested negative for CMT. **(B)** Detection of multiple pathogen structural patterns via tNGS.

Among 51 infectious cases with true-negative or false-positive CMT results, tNGS yielded true-negative or false-positive results in eight cases and true-positive results in 43 (84.3%). Of the latter, 55.8% were positive for viruses, followed by bacteria (46.5%) and fungi (18.6%) (**Figure 4**). The five most common microorganisms detected were human parainfluenza virus 3 (HPIV3) (nine cases), SARS-CoV-2 (eight cases), *S. aureus* (eight cases), *K. pneumoniae* (six cases) and *Candida* (five cases). Among the 15 (34.9%) polymicrobial infections, the most frequent pathogen was *S. aureus* (eight cases), followed by *K. pneumoniae* (five cases), *Candida* (three cases), and SARS-CoV-2 (three cases).

We finally retrospectively reviewed the antibiotic regimen. In 70 infectious cases with positive detection results for CMT, the overall treatments success rate (TSR) was 58.5% (n=41). For infectious cases with positive tNGS detection results, the TSR was 76.9% (80/104). For those cases with positive mNGS detection results, the TSR was 58.3% (14/24). Additionally, tNGS improved the overall treatment success rate by 69.7% (69/99 cases) in CMT true-negative or CMT-partially matched cases.

### Comparison of paired tNGS and mNGS tests

We next analysed the microorganisms detected with paired respiratory tNGS and mNGS (**Supplementary Figure S4**). Of note, RNA viruses, such as HPIV3, were only detected by tNGS. In the respiratory tNGS-blood mNGS pairs, 25 cases were diagnosed with infection. In 22 of these cases, tNGS results were consistent with the identified causative pathogens, yielding a concordance rate of 88%, while blood mNGS results matched the causative pathogens in only 40% of cases (*P* < 0.001).

**Figure 5:**
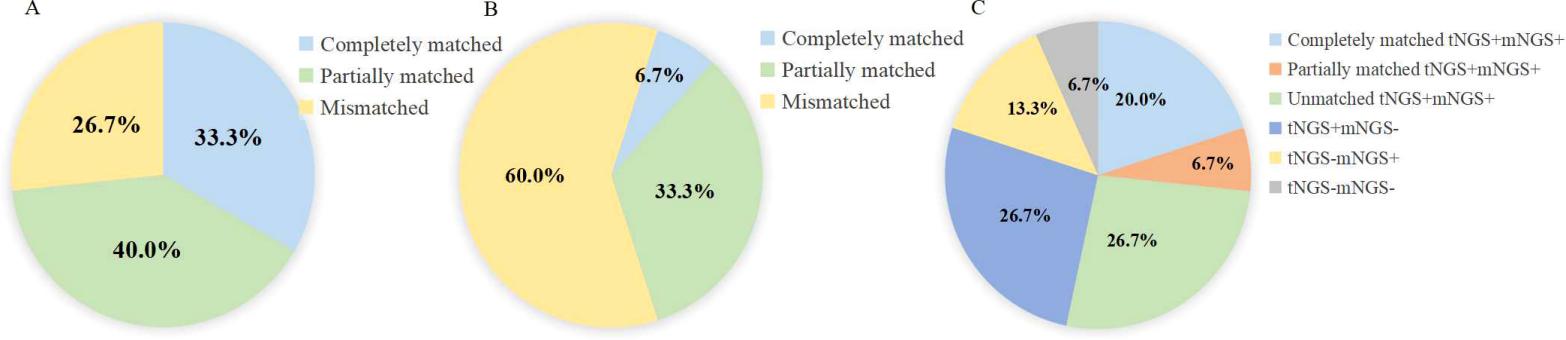
The concordance of pathogens identified by tNGS and mNGS in lower respiratory tract samples. **(A)** The concordance of pathogens identified by tNGS with causative pathogens. **(B)** concordance of pathogens identified by mNGS with causative pathogens. **(C)** Concordance of tNGS and mNGS results in respiratory samples.

In the respiratory tNGS-respiratory mNGS pairs [oropharyngeal swab tNGS-BALF mNGS (n=1), sputum tNGS-BALF mNGS (n=10), BALF tNGS- BALF mNGS (n=5), sputum tNGS- sputum mNGS (n=1), sputum tNGS- pleural effusion mNGS (n=1)], 15 cases were diagnosed with infection. tNGS results in 11 of these cases were consistent with the causative pathogens, resulting in a concordance rate of 73.3%, while respiratory mNGS results matched the causative pathogens in 40% of cases (*P* = 0.139). Specifically, the tNGS results were completely matched with the causative pathogens in 33.3% (5/15) of cases and partially matched in 40% (6/15) (**Figure 5a**). By contrast, the respiratory mNGS results were completely matched with the causative pathogens in only 6.7% (1/15) of cases, and partially matched in 33.3% (5/15) (**Figure 5b**).

We finally assessed the concordance between respiratory tNGS and respiratory mNGS results. For these cases, the results were completely consistent in 26.7% (4/15) of cases (both positive: 20%, both negative: 6.7%), partially consistent in 6.7% (1/15), and unmatched in 66.7% (10/15) cases (**Figure 5c**).

## Discussion

Intensive chemotherapy, as well as targeted therapy and HSCT, renders 27-50% of patients with haematologic malignancies susceptible to respiratory infections (Specchia et al., 2003; Periselneris and Brown, 2019). Traditional approaches for culturing microbes for pathogen detection are time-consuming and have low sensitivity (Duan et al., 2021). Although mNGS is an emerging method for causative pathogen detection in immunodeficient patients, this approach is limited by compromised sensitivity caused by the high DNA background of the human host (Chiu and Miller, 2019). tNGS, based on ultra-multiplex PCR and high-throughput sequencing, offers distinct advantages in terms of the cost and detection process, with a quarter of the cost of mNGS and the ability of simultaneously detecting multiple pathogens (Gaston et al., 2022; Deng et al., 2023). Nevertheless, the significance of tNGS performance in immunocompromised patients remains unclear. Here, we showed that tNGS was superior to CMT for detecting pathogens, especially viruses and *Pneumocystis jirovecii*. Diagnostic approaches for respiratory viruses have relied on direct fluorescent antigen assays and multiplex PCR panels, but the number of suitable monoclonal antibodies is limited. Meanwhile, nucleic acid amplification via quantitative PCR can only target a single pathogen, with high loads for each examination. In our hospital’s diagnostic laboratory, PCR-based detection of viral pathogens is performed as follows: HSV1, herpes simplex virus-2, and EBV can be identified from throat swab specimens, while CMV detection through PCR is available for other clinical secretions though subject to sample type limitations. By contrast, tNGS technology allows the simultaneous amplification of various respiratory viruses (Fox, 2007). Indeed, Dai et al (Dai et al., 2022). previously proposed that tNGS could increase the pathogen detection rate by approximately 40% compared to traditional culture-based approaches for respiratory infectious diseases. Consistently, we found that tNGS was more efficient than CMT even for rare pathogen detection, as tNGS identified the specific pathogen *Mycobacterium* in one case, and *Nocardia* in two cases.

The sensitivity of CMT is limited by the culture conditions required for certain pathogens and the time involved (Gu et al., 2019; Shi et al., 2020). In cases negative by CMT, tNGS improved detection efficiency, with a pathogen detection rate of 84.3%, particularly for the diagnosis of polymicrobial infections (36.6%). *Staphylococcus aureus*, *Klebsiella pneumoniae*, *Candida*, and SARS-CoV-2 were the most commonly detected pathogens in our study. tNGS thus proves highly valuable for detecting highly pathogenic bacteria and fungi, facilitating rapid and accurate treatment. Moreover, compared with CMT, the sensitivity of tNGS was significantly greater (87.7% vs. 52.5%; P < 0.001).

The respiratory tract is replete with diverse microbial communities. Indeed 90/120 immunosuppressed patients in our study cohort were found by tNGS to be colonized by microbial communities. Consistent with findings in healthy controls, the prominent genera were gram-negative *Prevotella*, *Veillonella*, and gram-positive *Streptococcus* and *Pseudomonas*, which maintain pulmonary immune homeostasis (Dickson et al., 2013). The bacterial colonizing communities consisted of *Streptococcus*, *Micromonas*, *Fusobacterium*, and *Acinetobacter*, with a colonization percentage of 23.3%. *Candida* species (found in the respiratory tract of 9.2% of patients) are normal inhabitants of the human mucosa, but their prevalence increases in immunocompromised hosts (Koltsida and Zaoutis, 2021). EBV was the most prevalent microecological pathogen, with a prevalence of 48.3% in patients versus only 20% in healthy controls (Chen et al., 2020). A unique observation in immunocompromised hosts was that human herpesvirus 7 colonized the respiratory tract (40.8%). We suspect, therefore, that in immunocompromised populations, the respiratory microecology is typically characterized by an increase in viral/fungal colonization and a decrease in bacterial colonization.

In the comparison of mNGS and tNGS, tNGS provided superior diagnostic ability with greater sensitivity than mNGS for all specimens (82.5% *vs.* 40%) and coincident specificity (40%). It is important to note that all samples used for the tNGS assay were derived from the respiratory tract, whereas 59.1% of the samples for mNGS were from peripheral blood, and 40.9% were from the respiratory tract. Nevertheless, our hypothesis that tNGS has promising diagnostic potential over mNGS in respiratory tract infection was confirmed by the fact that the tNGS results matched the causative pathogens at the species level significantly better than blood mNGS results (88% *vs.* 40%, *P* < 0.001) and slightly better than respiratory tract mNGS results (73.3% *vs.* 40%, *P* = 0.139). A previous study evaluating mNGS in pneumonia patients demonstrated that mNGS with BALF samples is more sensitive than with blood samples for pathogen detection, especially for bacteria and fungi (Chen et al., 2020). mNGS, based on untargeted shotgun sequencing, theoretically has the capacity to detect a greater abundance of pathogens than tNGS (Chiu and Miller, 2019).tNGS, however, is capable of detecting > 95% of respiratory pathogens (Li et al., 2022), with the added advantages of lower cost and shorter turnaround time compared to mNGS (Huang et al., 2023).

There are several limitations to consider when interpreting the results of this study. First, we were unable to recruit the appropriate patients for inclusion to determine the optimal threshold of colonizing microorganisms for tNGS. Second, the tNGS tests is regarded as the index test in this study. Although the final diagnosis did not depend on the tNGS results, the causative pathogens was evaluated with the reference of the NGS findings, especially in the instance of negative CMT results. The interpretation of pathogen reports derived from tNGS is challenging. The tNGS panel we utilized encompasses 198 pathogens, which typically results in preliminary reports that include various microorganisms, such as pathogenic and opportunistic pathogens, as well as colonizing microbiota. The discrepancies between colonizing or background microorganisms and infectious pathogens are ambiguous. Due to the absence of standardized protocols (see **Supplementary Table S1**), our interpretation of tNGS results is based on classifying pathogens according to specimen type, which may influence the occurrence of false-positive and false-negative results. This could lead to an overestimation of tNGS diagnostic accuracy. In addition, TSR in this study referred to the index test (tNGS), which did not align with QUADAS-2 guidelines (Whiting et al., 2011) and introduce incorporation bias. Third, considering infection possibility of microorganisms with moderate pathogenic ability, pathogen reads should be given more attention. We initially assessed these microorganisms as B type in the primary report and they were further verified as infectious pathogens after consideration by the committee as mentioned above. Finally, prospective multicentre studies are now warranted to verify the effectiveness of tNGS and overcome the inherent limitations of a retrospective analysis.

## Conclusions

Our retrospective analysis showed that tNGS has equal diagnostic potential to mNGS in identifying clinically relevant pathogens in patients with suspected respiratory infections, with a higher degree of accuracy for highly pathogenic and novel microorganisms. tNGS provides valuable information to optimize antibiotic therapy in CMT-negative cases and thus stands to reduce the abuse of anti-infective drugs and improve survival rate. Further research to verify the validity of tNGS as a routine screening method for patients with a suspected respiratory infection is now warranted.

## Data Availability

The raw data supporting the conclusions of this article will be made available by the authors, without undue reservation.
