# Supplementary material for: Targeted next-generation sequencing for respiratory infections in patients with haematological malignancies
Source: Front Cell Infect Microbiol. 2025 Nov 19;15:1678858. doi: 10.3389/fcimb.2025.1678858 (PMC12672896; doi:10.3389/fcimb.2025.1678858)
Supplement: Supplementary file 1 [file DataSheet1.docx]

**Supplementary Materials**

**Application of targeted next-generation sequencing for respiratory infections in patients with hematological malignancies**

Jiayu Huang, Su Li, Chuanhe Jiang, Luxiang Wang, Zengkai Pan, Zilu Zhang, Jun Zhu, Wei Chen, Xiaoxia Hu

**Corresponding author:** Xiaoxia Hu: [hu_xiaoxia@126.com](mailto:hu_xiaoxia@126.com)

State Key Laboratory of Medical Genomics, Shanghai Institute of Hematology, National Research Center for Translational Medicine, Shanghai Rui Jin Hospital, Shanghai Jiao Tong University School of Medicine, Shanghai, China

**Supplemental Fig. S1: Flow chart of the study.**


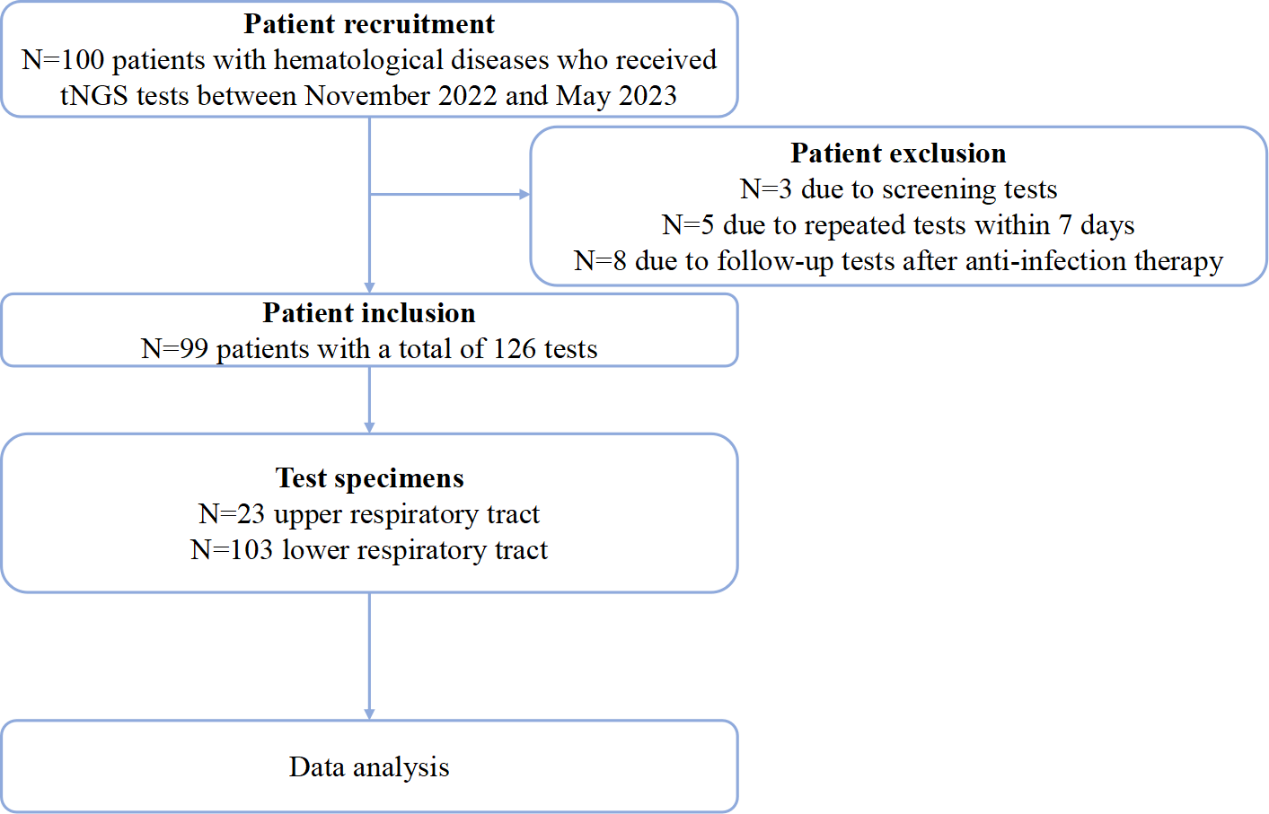


**Supplemental Fig. S2: Distribution of the detected pathogens in the two groups.** Group I: upper respiratory tract, Group II: lower respiratory tract.


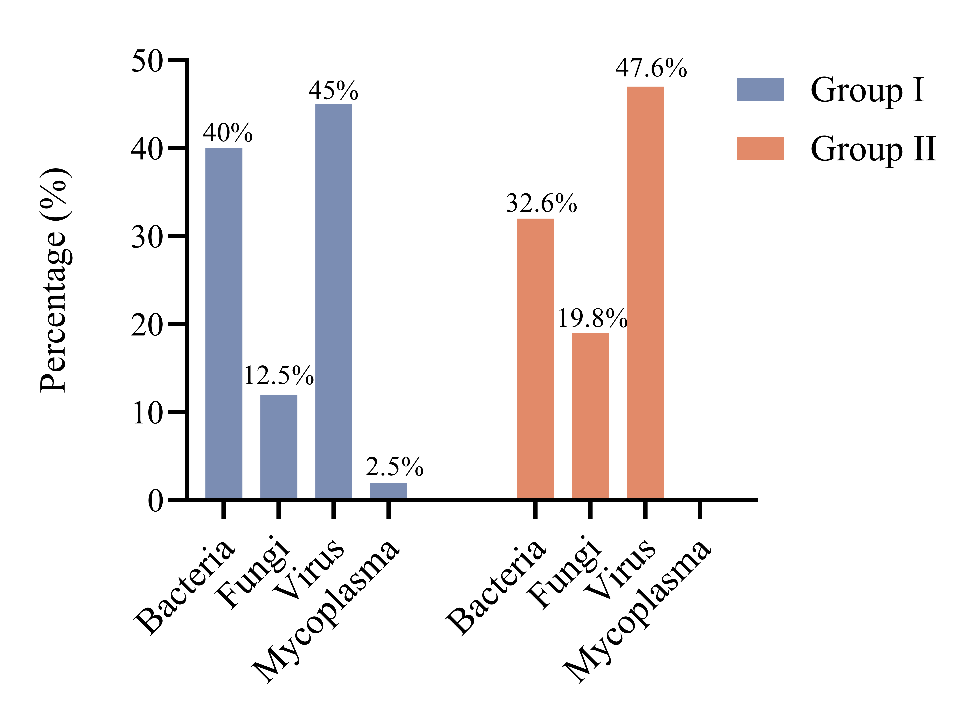


**Supplemental Fig. S3: Micro-organisms detected via tNGS and CMT.** (A) Specific micro-organism. (B) Comparison of the micro-organism detection rate. ***, *P* <0.001; #, *P*>0.05

Abbreviations: HSV1, herpes simplex virus-1; HHV6B, Human herpesvirus 6B; B19, human parvovirus B19; HPIV3, Human parainfluenza virus 3; RSV, Respiratory syncytial virus; EBV, Epstein-barr virus; HHV7, Human herpesvirus 7; CMV, Cytomegalovirus; HHV6, Human herpesvirus 6; VZV, Varicella-zoster virus; and HBoV1, Human Bocavirus 1.


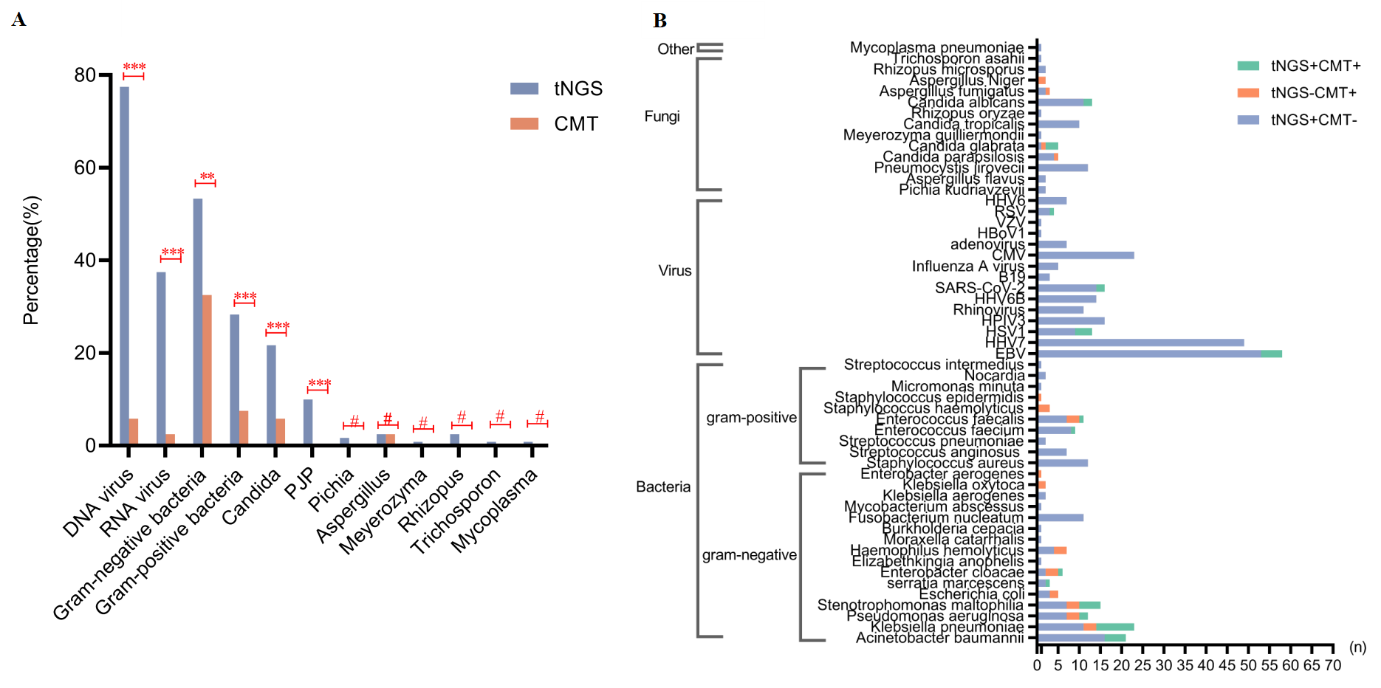


**Supplemental Fig. S4: Micro-organism map for lower respiratory tract specimens analyzed by tNGS and mNGS.** Abbreviations: HSV1, herpes simplex virus-1; HHV6, Human herpesvirus 6; HHV7, Human herpesvirus 7; CMV, Cytomegalovirus; HBoV1, Human Bocavirus 1; HHV6B, Human herpesvirus 6B; EBV, Epstein-barr virus; B19, human parvovirus B19; HPIV3, Human parainfluenza virus 3; and TTV: Torque teno virus.


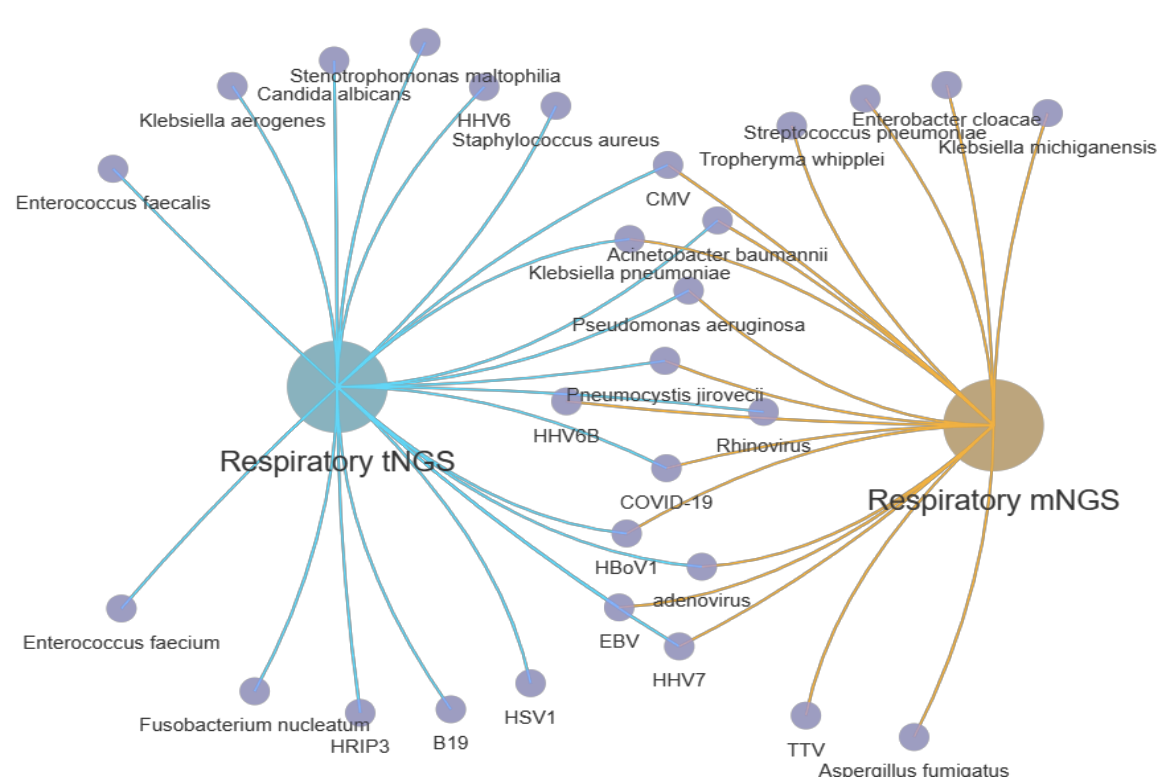


**Supplemental Table S1: Targeted microorganisms’ classification by specimens.**

| Categories | | Species | Pathogens |
| --- | --- | --- | --- |
| Bacteria | G+ | Corynebacterium | Corynebacterium diphtheriae |
|  |  | Mycobacterium | Mycobacterium asiaticum, Mycobacterium avium, Mycobacterium avium complex, Mycobacterium celatum, Mycobacterium gordonae,Mycobacterium kansasii, Mycobacterium intracellulare, Mycobacterium malmoense, Mycobacterium nontuberculosis, Mycobacterium scrofulaceum, Mycobacterium shimoidei, Mycobacterium simiae, Mycobacterium szulgai, Mycobacterium tuberculosis complex, Mycobacterium xenopi, Mycolicibacterium fortuitum, Mycolicibacterium smegmatis |
|  |  | Mycobacteroides | Mycobacteroides abscessus, Mycobacteroides abscessus complex,  Mycobacteroides chelonae |
|  |  | Nocardia | Nocardia abscessus, Nocardia africana, Nocardia asteroides, Nocardia brasiliensis,  Nocardia concava, Nocardia cyriacigeorgica, Nocardia farcinica, Nocardia nova,  Nocardia otitidiscaviarum, Nocardia terpenica |
|  |  | Parvimonas | Parvimonas micra |
|  |  | Rhodococcus | Rhodococcus hoagii |
|  |  | Staphylococcus | Staphylococcus aureus |
|  |  | Streptococcus | Streptococcus agalactiae, Streptococcus anginosus group, Streptococcus intermedius, Streptococcus pneumoniae, Streptococcus pyogenes |
|  |  | Tropheryma | Tropheryma whipplei |
|  |  | Trueperella | Trueperella pyogenes |
|  | G- | Acinetobacter | Acinetobacter baumannii, Acinetobacter junii, Acinetobacter ursingii |
|  |  | Bacteroides | Bacteroides fragilis |
|  |  | Bordetella | Bordetella holmesii, Bordetella parapertussis, Bordetella pertussis |
|  |  | Brucella | Brucella |
|  |  | Burkholderia | Burkholderia cenocepacia, Burkholderia cepacia, Burkholderia cepacia complex, Burkholderia mallei, Burkholderia pseudomallei, Burkholderia multivorans |
|  |  | Elizabethkingia | Elizabethkingia anophelis, Elizabethkingia meningoseptica |
|  |  | Enterobacter | Enterobacter cloacae complex |
|  |  | Escherichia | Escherichia coli |
|  |  | Fusobacterium | Fusobacterium necrophorum, Fusobacterium nucleatum |
|  |  | Haemophilus | Haemophilus influenzae |
|  |  | Klebsiella | Klebsiella aerogenes, Klebsiella oxytoca, Klebsiella pneumoniae, Klebsiella variicola |
|  |  | Legionella | Legionella pneumophila, Legionella bozemanii, Legionella longbeachae, Legionella lmicdadei |
|  |  | Moraxella | Moraxella catarrhalis |
|  |  | Neisseria | Neisseria meningitidis |
|  |  | Pasteurella | Pasteurella multocida |
|  |  | Proteus | Proteus mirabilis |
|  |  | Pseudomonas | Pseudomonas aeruginosa |
|  |  | Serratia | Serratia marcescens |
|  |  | Stenotrophomonas | Stenotrophomonas maltophilia |
| Fungi | Fungi | Nakaseomyces | [Candida] glabrata |
|  |  | Aspergillus | Aspergillus flavus complex, Aspergillus fumigatus, Aspergillus niger complex, Aspergillus terreus complex |
|  |  | Candida | Candida albicans, Candida orthopsilosis, Candida parapsilosis, Candida tropicalis |
|  |  | Cryptococcus | Cryptococcus gattii, Cryptococcus neoformans |
|  |  | Fusarium | Fusarium |
|  |  | Histoplasma | Histoplasma capsulatum |
|  |  | Lichtheimia | Lichtheimia ramosa, Lichtheimia corymbifera |
|  |  | Meyerozyma | Meyerozyma guilliermondii |
|  |  | Mucor | Mucor irregularis, Mucor racemosus |
|  |  | Pichia | Pichia kudriavzevii |
|  |  | Pneumocystis | Pneumocystis jirovecii |
|  |  | Rhizomucor | Rhizomucor pusillus |
|  |  | Rhizopus | Rhizopus delemar, Rhizopus microsporus, Rhizopus oryzae |
|  |  | Scedosporium | Scedosporium apiospermum,Scedosporium boydii |
|  |  | Talaromyces | Talaromyces marneffei |
|  |  | Trichosporon | Trichosporon asahii |
| Virus | DNA | Betapolyomavirus | Human polyomavirus 1, JC polyomavirus, WU Polyomavirus |
|  |  | Mastadenovirus | Human adenovirus(1, 2, 21, 5, 6, 7, B3, E4, 55, 57, 11, 14, 34, 35), Human adenovirus B, Human adenovirus C, Human mastadenovirus D,Human adenovirus E |
|  |  | Simplexvirus | Human alphaherpesvirus (1, 2) |
|  |  | Varicellovirus | Human alphaherpesvirus 3 |
|  |  | Cytomegalovirus | Human betaherpesvirus 5 |
|  |  | Roseolovirus | Human herpes virus (6A, 6B, 6, 7) |
|  |  | Bocaparvovirus | Human bocavirus (1, 2, 3, 4) |
|  |  | Lymphocryptovirus | Human gammaherpesvirus 4 |
|  |  | Erythroparvovirus | Human parvovirus B19 |
|  | RNA | Enterovirus | Coxsackievirus (A10, A16, A2, A5, A6, B3), Echovirus (E18, E30), Enterovirus (A, A71, B, C, D, D68), Rhinovirus (A, B, C) |
|  |  | Alphacoronavirus | Human coronavirus(229E, NL63) |
|  |  | Metapneumovirus | Human metapneumovirus |
|  |  | Rubulavirus | Human rubulavirus (2, 4) |
|  |  | Orthopneumovirus | Human respiratory syncytial virus (A, B), |
|  |  | Respirovirus | Human respirovirus (1, 3) |
|  |  | Alphainfluenzavirus | Influenza A virus(H1N1, H3N2, H5N1, H7N9, H1N1pdm09) |
|  |  | Betainfluenzavirus | Influenza B virus(B/Victoria, B/Yamagata) |
|  |  | Gammainfluenzavirus | Influenza C virus |
|  |  | Morbillivirus | Measles morbillivirus |
|  |  | Orthorubulavirus | Mumps orthorubulavirus |
|  |  | Rubivirus | Rubella virus |
|  |  | Betacoronavirus | Human coronavirus(HKU1,OC43), SARS-CoV-2 |
| Other | Other | Mycoplasma | Mycoplasma pneumoniae |
|  |  | Ureaplasma | Ureaplasma parvum, Ureaplasma urealyticum |
|  |  | Chlamydia | Chlamydia pneumoniae, Chlamydia trachomatis, Chlamydia psittaci |
|  |  | Coxiella | Coxiella burnetii |

Abbreviations: G-, Gram-negative; and G+, Gram-positive.

**Supplemental Table S2: tNGS false-positive, false-negative cases.**

| **Types** | | | **Pathogens** |
| --- | --- | --- | --- |
| tNGS missed clinically significant infections (14 cases) | False negative (10 cases) | Colonization (7 cases) | EBV |
|  |  |  | HHV7 |
|  |  |  | Acinetobacter baumannii |
|  |  | Negative (3 cases) | NA |
|  | False positive (4 cases) | Not consistent with clinical diagnosis (4 cases) | Staphylococcus aureus |
|  |  |  | mucosal Rhinovirus |
| tNGS erroneously classified cases as infections (4 cases) | False positive (4 cases) | Likelihood of latent infection (3 cases) | CMV |
|  |  |  | Adenovirus C |
|  |  |  | Rhinovirus |
|  |  | potential concomitant infection with immune events (1 case) | Klebsiella pneumoniae |

Abbreviations: EBV, Epstein-Barr virus; CMV, Cytomegalovirus; HHV7, Human herpes virus-7; and tNGS, targeted next-generation sequencing.
